# Evaluation of a Booster Dose of Pentavalent Rotavirus Vaccine Coadministered With Measles, Yellow Fever, and Meningitis A Vaccines in 9-Month-Old Malian Infants

**DOI:** 10.1093/infdis/jiy215

**Published:** 2018-04-12

**Authors:** Fadima C Haidara, Milagritos D Tapia, Samba O Sow, Moussa Doumbia, Flanon Coulibaly, Fatoumata Diallo, Awa Traoré, Mamoudou Kodio, Corey L Kelly, Meagan Fitzpatrick, Karen Kotloff, John C Victor, Kathleen Neuzil

**Affiliations:** 1 Centre pour le Développement des Vaccins–Mali, Bamako; 2 Center for Vaccine Development, University of Maryland School of Medicine, Baltimore; 3 PATH, Seattle, Washington

**Keywords:** Rotavirus vaccine, Mali, infants, booster dose, yellow fever vaccine, measles vaccine

## Abstract

**Background:**

Rotavirus vaccines given to infants are safe and efficacious. A booster dose of rotavirus vaccine could extend protection into the second year of life in low-resource countries.

**Methods:**

We conducted an open-label, individual-randomized trial in Bamako, Mali. We assigned 600 infants aged 9–11 months to receive measles vaccine (MV), yellow fever vaccine (YFV), and meningococcal A conjugate vaccine (MenAV) with or without pentavalent rotavirus vaccine (PRV). We assessed the noninferiority (defined as a difference of ≤10%) of seroconversion and seroresponse rates to MV, YFV, and MenAV. We compared the seroresponse to PRV.

**Results:**

Seroconversion to MV occurred in 255 of 261 PRV recipients (97.7%) and 246 of 252 control infants (97.6%; difference, 0.1% [95% confidence interval {CI}, −4.0%–4.2%]). Seroresponse to YFV occurred in 48.1% of PRV recipients (141 of 293), compared with 52.2% of controls (153 of 293; difference, −4.1% [95% CI, −12.2%–4.0%]). A 4-fold rise in meningococcus A bactericidal titer was observed in 273 of 292 PRV recipients (93.5%) and 276 of 293 controls (94.2%; difference, −0.7% [95% CI, −5.2%–3.8%]). Rises in geometric mean concentrations of immunoglobulin A and immunoglobulin G antibodies to rotavirus were higher among PRV recipients (118 [95% CI, 91–154] and 364 [95% CI, 294–450], respectively), compared with controls (68 [95% CI, 50–92] and 153 [95% CI, 114–207], respectively).

**Conclusions:**

PRV did not interfere with MV and MenAV; this study could not rule out interference with YFV. PRV increased serum rotavirus antibody levels.

**Clinical Trials Registration:**

NCT02286895.

## Teaser

Receipt of pentavalent rotavirus vaccine at 9 months of age in Mali increased antibody levels and did not interfere with responses to measles vaccine and meningococcal A conjugate vaccine. Interference with yellow fever vaccine could not be ruled out.

Rotavirus is the most common cause of severe and fatal diarrhea in young children throughout the world [1, 2]. The importance of rotavirus disease is well established in the first year of life, and studies in low-resource settings demonstrate that severe rotavirus incidence remains high through the second year of life [2, 3]. Oral rotavirus vaccines are currently recommended by the World Health Organization (WHO) Strategic Advisory Group of Experts. While rotavirus vaccines reduce severe rotavirus disease in the first year of life in low-resource settings, declines in efficacy have been reported in the second year of life [3–7].

Improving protection beyond that achieved with the current vaccination schedule could have significant global impact. One strategy to extend protection is to administer a booster dose of rotavirus vaccine at 9 months of age, concomitant with other routinely recommended Expanded Program on Immunization vaccines. In a study in Bangladesh, human monovalent rotavirus vaccine administered at 9 months of age did not interfere with immune responses to concomitantly administered vaccines and significantly increased rates of seropositivity for immunoglobulin A (IgA) and immunoglobulin G (IgG) antibodies to rotavirus [8]. Moreover, a recent model estimates that up to 20000 additional deaths could be averted with a booster dose administered at 9–12 months of age in countries with moderate and high mortality rates among children [9].

A booster dose of rotavirus vaccine had never previously been evaluated in African infants, nor were data available for the pentavalent rotavirus vaccine (PRV). In 2014, Mali introduced PRV into their routine immunization program for infants aged 6, 10, and 14 weeks. To assess the effect of concomitant administration of PRV on measles vaccine (MV) and yellow fever vaccine (YFV), we compared immune responses to these vaccines in Malian infants receiving a supplemental dose of PRV to those observed in the absence of PRV (control group). We also measured antirotavirus immune responses within both groups. Finally, since meningococcal A conjugate vaccine (MenAV) was planned to be added to the Expanded Program on Immunization schedule at the same age, we characterized immune responses to that vaccine in the presence and absence of PRV.

## METHODS

### Study Design and Participants

We conducted an open-label, individual-randomized, comparative immunogenicity trial. From 15 October 2014 to 18 December 2014, participants were enrolled at 9 health centers in Bamako, Mali. Eligible infants were aged 9–11 months; resided in the study area; were generally healthy; had been fully vaccinated according to the local immunization schedule, as verified by review of the vaccination record; and had parents who were willing to follow protocol procedures. Infants were ineligible if they had history of any of the following: prior receipt of MV, YFV, or MenAV; receipt of rotavirus vaccine in the past 90 days; known hypersensitivity to any component of the study vaccines and/or following administration of previous vaccines; and any chronic medical condition or medications that might compromise the well-being of the participant, compromise compliance with study procedures, or interfere with the outcome of the study. Moderate or severe acute illness at the time of enrollment was a temporary exclusion at the discretion of the investigator.

The protocol was approved by the University of Maryland School of Medicine Institutional Review Board; the Ethical Committee of Faculté de Médecine, Pharmacie, et Odontostomatologie of Mali; the Ministry of Health of Mali; Western Institutional Review Board (Puyallup, WA); and leaders of the involved communities. Parents or guardians of participants provided informed consent prior to initiation of study procedures. The trial was registered with ClinicalTrials.gov (NCT02286895).

### Procedures

All participants received MV (Serum Institute of India) by subcutaneous injection, YFV (Federal State Unitary Enterprise on Manufacture of Bacterial and Viral Preparations, Chumakov Institute of Poliomyelitis and Viral Encephalitides, Russian Academy of Medical Sciences) by intramuscular injection, and MenAV 5 μg (Serum Institute of India) by intramuscular injection at a separate site. Participants were randomly assigned at a ratio of 1:1 by a sequentially assigned numeric code to receive oral PRV (Merck) or no PRV. Placebo was not used in this study because PRV is used by the public-sector immunization program in Mali and because no placebo could be accessed. Laboratory staff performing serologic tests remained masked to individual participant group assignments.

All participants were observed for 30 minutes after vaccination. Local reactions (induration and pain) and systemic reactions (fever, lethargy, irritability, vomiting, diarrhea, loss of appetite, rash, persistent crying, and signs of potential intussusception) were assessed on days 1–5 during home visits by the trained field workers and on day 7 (±1 day) by a physician in clinic. Unsolicited adverse events (AEs) were assessed until day 28. Serious AEs were assessed from day 0 until the end of the study (day 84).

Blood samples (3–5 mL) were collected on days 0 and 28 for evaluation of antibody responses to all antigens. The anti–measles virus antibody titer was determined by a commercially available enzyme-linked IgG immunoassay (Wampole Laboratories, Princeton, NJ). Yellow fever virus–specific neutralizing antibody titers (NTs) were determined by use of the Robert Koch Institute’s standard operating procedure and with respect to international scientific references [10, 11]. A validated serum bactericidal assay (SBA) that uses baby rabbit complement was used to measure the titer of functional antibody in human sera to *Neisseria meningitidis* group A [12, 13]. Anti–rotavirus IgA and IgG levels were measured by an enzyme-linked immunosorbent assay at the Laboratory of Specialized Clinical Studies at the Cincinnati Children′s Hospital Medical Center (Cincinnati, OH), as described previously [14–16]. The positive control was pooled sera from subjects who had received a rotavirus vaccine or had experienced a natural rotavirus infection. The negative control was sera shown to have no antibody to rotavirus.

### Outcomes

The study had 2 coprimary outcomes. First, we determined the noninferiority of the anti–measles virus IgG seroconversion rate 28 days after vaccination in the PRV group as compared to that in the group without PRV. Seroconversion was defined as seropositivity at day 28 among participants who were seronegative at baseline (defined as a titer of ≤0.90). Second, we evaluated the noninferiority of the yellow fever virus NT response in the 2 groups. A ≥4-fold response in the postvaccination yellow fever virus NT as compared to the prevaccination yellow fever virus NT, regardless of baseline serostatus, was used to define seroresponse to vaccination.

We evaluated additional evidence of PRV interference with the immune responses to MV, YFV, and MenAV by comparing the difference between PRV and control groups with regard to the following secondary outcomes: anti–measles virus IgG seroconversion rates at day 84; the yellow fever virus geometric mean titers (GMTs) at day 28; yellow fever virus seroresponse rate (defined as a ≥2-fold increase in the NT, compared with baseline) at day 28; yellow fever virus NT seroconversion rate (defined as a positive result [ie, a titer ≥1:8] among those seronegative at baseline) at day 28; SBA seroresponse rate (defined as a ≥4-fold increase in the titer, compared with baseline) at day 28; SBA GMT at day 28; and anti–rotavirus IgA and IgG seroresponse rates at day 28, restricted to those with baseline levels <20 U/mL in each group. We also conducted a superiority evaluation of the ratio of anti–rotavirus IgA and IgG geometric mean concentrations (GMCs) in the PRV group versus the control group at day 28, first inclusive of all participants and then restricted only to subjects with baseline levels <20 U/mL. Secondary outcomes related to safety were descriptive and included the proportion of participants in each group with any of the following: immediate reactions occurring in the first 30 minutes after vaccination, solicited adverse reactions, AEs, or SAEs.

### Statistical Analysis

All immunogenicity analyses and summaries were performed on a per-protocol basis (definition for each outcome are specified below). Supportive intention-to-treat analyses were conducted on all enrolled participants who received at least 1 dose of study vaccine. Safety analyses were conducted on this same intention-to-treat basis.

For the measles outcome, the per-protocol cohort included infants meeting all inclusion and no exclusion criteria, having less than seroprotective levels of measles virus IgG before vaccination, and receiving study vaccines and undergoing blood specimen collection according to schedule. Yellow fever virus–associated secondary analyses were conducted on the same per-protocol cohort, except that infants were required to have less than seroprotective anti–yellow fever virus NTs before vaccination instead of measles virus antibody.

For the MV and YFV immunogenicity primary analyses, proportions of participants reaching seroprotective levels and NTs of measles virus IgG levels and yellow fever virus, respectively, at prespecified time points after vaccination were compared between groups, using the Newcombe-Wilson method without continuity correction. A noninferiority margin of −10% was chosen as the maximal absolute reduction in proportion allowed in the group that received concomitant MV, YFV, MenAV, and PRV as compared to the group that received MV, YFV, and MenAV. Rotavirus and meningococcus A immunogenicity analyses were conducted on the per-protocol cohort without the requirement for less than seroprotective levels of measles virus or yellow fever virus IgG levels and NTs, respectively, before vaccination. Anti–rotavirus IgA and IgG GMCs and the proportion of infants who were seropositive were compared before and after vaccination in each group, using the McNemar test for correlated proportions; for GMC calculations, concentrations of <20 U/mL were converted to 10 U/mL.

We assumed 90% seroconversion and seroresponse rates in each arm for each antigen in the 2 coprimary objectives. For each coprimary outcome, to rule out a noninferiority margin of ≤10% with 95% power and a 1-sided type 1 error rate of ≤2.5%, 237 evaluable subjects were required in each group (a power of 95% was chosen to give an overall power of at least 90%.) On the assumption that 79% of the cohort could be evaluated (based on a baseline seropositivity rate of 10% and a loss to follow-up of 12%), a sample size of 600 vaccinated subjects was required.

## RESULTS

**Figure 1.**
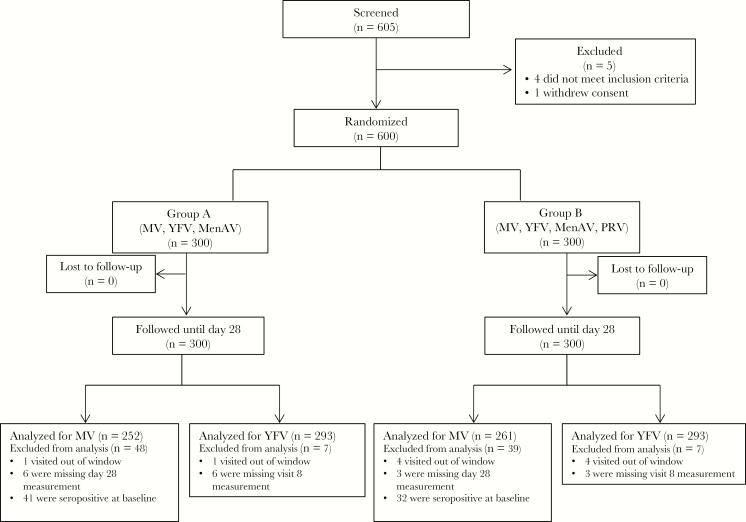
Study profile. MenAV, meningococcal A conjugate vaccine; MV, measles vaccine; PRV, pentavalent rotavirus vaccine; YFV, yellow fever vaccine.

From 15 October 2014 to 18 December 2014, 605 infants were screened and 600 enrolled with 300 receiving PRV. All participants completed all study visits until 5 months after vaccination (Figure 1). Study follow-up was completed on 23 March 2015. The baseline characteristics among participants in both study groups were similar (Table 1).

**Table 1.** Baseline Characteristics of Vaccinated Participants, by Vaccines Received—Per-Protocol Population

Characteristic	MV, YFV, MenAV, and PRV (n = 300)	MV, YFV, and MenAV (n = 300)
Age, mo	9.7 ± 0.7	9.7 ± 0.7
Male sex	149 (49.7)	167 (55.7)
Length, cm	69.5 ± 2.5	69.6 ± 2.6
Weight, kg	8.1 ± 1.0	8.2 ± 1.0

Data are mean value ± SD or no. (%) of infants.

Abbreviations: MenAV, meningococcal A conjugate vaccine; MV, measles vaccine; PRV, pentavalent rotavirus vaccine; YFV, yellow fever vaccine.

### Measles Virus

**Figure 2.**
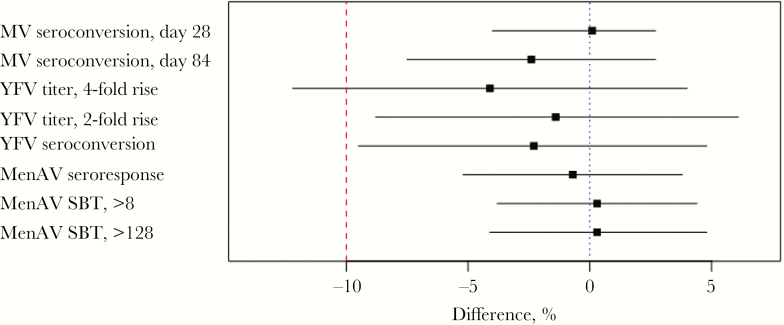
Noninferiority of immune responses to concomitantly administered vaccines between recipients of measles vaccine (MV), yellow fever vaccine (YFV), meningococcal A conjugate vaccine (MenAV), and pentavalent rotavirus vaccine (PRV) and recipients of MV, YFV, and MenAV—per-protocol population. The dotted line indicates no difference, and the dashed line indicates the noninferiority margin of 10%. SBT, serum bactericidal titer .

Before vaccination, 85.5% participants (87% of PRV recipients and 84% of controls) were seronegative for measles virus. Seropositivity was observed 28 days after vaccination in 255 of 261 PRV recipients (97.7%) and 246 of 252 controls (97.6%), for a difference of 0.1% (95% CI, −4.0%–4.2%) in the seroconversion rate (Table 2). On day 84, 210 of 228 PRV recipients (92.1%) who seroconverted remained seropositive, compared with 206 of 218 controls (94.5%), for a difference of −2.4% (95% CI, −7.5%–2.7%; Table 2). Similar results were obtained in the intention-to-treat population (data not shown). These results met our prespecified criteria for noninferiority of the response to MV (Figure 2).

**Table 2.** Anti–Measles Virus Immunoglobulin G Seroconversion Rates in Infants, by Vaccines Received—Per-Protocol Population

Seroconversion,^a^ Time Point	MV, YFV, MenAV, and PRV		MV, YFV, and MenAV		Difference, Percentage Points (95% CI)
	Proportion	Percentage (95% CI)	Proportion	Percentage (95% CI)	
Day 28	255/261	97.7 (95.9–99.5)	246/252	97.6 (95.7–99.5)	0.1 (−4.0–4.2)
Day 84	210/228	92.1 (88.6–95.6)	206/218	94.5 (91.5–97.5)	−2.4 (−7.5–2.7)

Abbreviations: CI, confidence interval; MenAV, meningococcal A conjugate vaccine; MV, measles vaccine; PRV, pentavalent rotavirus vaccine; YFV, yellow fever vaccine.

^a^Defined as a positive response among those who were seronegative at baseline.

### Yellow Fever Virus

At baseline, YFV-specific immune responses were assessed in 586 participants. Increases of ≥4-fold in yellow fever virus plaque-reduction NTs occurred in 141 of 293 PRV recipients (48.1%) and 153 of 293 controls (52.2%), for a difference of −4.1% (95% CI, −12.2%–4.0%; Table 3). Because the lower bound on the CI of the difference exceeds −10, our primary criterion to establish the noninferiority of the response to YFV was not met (Figure 2). When seroresponse was defined as a ≥2-fold rise in NT, the response among PRV recipients (202 of 293 [68.9%]) was noninferior to that among controls (206 of 293 [70.3%]; difference, −1.4% [95% CI, −8.8%–6.1%]; Table 3 and Figure 2). Furthermore, seroconversion among participants who were seronegative at baseline (ie, those with a titer of <1:8) was comparable in both groups (difference, −2.3% [95% CI, −9.5%–4.8%; Table 3). Similar results were observed in the intention-to-treat population (data not shown). The GMTs were similar in both groups, and the ratio of the day 28 yellow fever virus GMT in the PRV group to that in the control group was 0.92 (95% CI, .77–1.09; *P* = .3150).

**Table 3.** Yellow Fever Virus Serostatus and Geometric Mean Plaque-Reduction Neutralizing Titers (GMTs) in Infants, by Vaccines Received—Per-Protocol Population

Variable	MV, YFV, MenAV, and PRV		MV, YFV, and MenAV		Difference, Percentage Points (95% CI)
	Proportion	Percentage (95% CI)	Proportion	Percentage (95% CI)	
Serologic finding					
Seroresponse^a^					
≥4-fold increase	141/293	48.1 (42.4–53.8)	153/293	52.2 (46.5–57.9)	−4.1 (−12.2–4.0)
≥2-fold increase	202/293	68.9 (63.6–74.2)	206/293	70.3 (65.1–75.5)	−1.4 (−8.8–6.1)
Seroconversion^b^	210/287	73.2 (68.0–78.3)	219/290	75.5 (70.6–80.5)	−2.3 (−9.5–4.8)
Time point	GMT (95% CI)		GMT (95% CI)		GMT Ratio (95% CI)
Baseline	2.49 (2.37–2.62)		2.40 (2.31–2.49)		…
Day 28	15.03 (13.31–16.97)		16.82 (14.80–19.11)		0.92^c^ (.77–1.09)

Abbreviations: CI, confidence interval; MenAV, meningococcal A conjugate vaccine; MV, measles vaccine; PRV, pentavalent rotavirus vaccine; YFV, yellow fever vaccine.

^a^Defined as the indicated fold increase in titer from baseline to day 28.

^b^Defined as seropositivity (titer, ≥1:8) at day 28 among participants who were seronegative (titer, <1:8) at baseline.

^c^ *P* = .3150.

### Rotavirus

Anti–rotavirus IgA and IgG seroconversion and seroresponse rates were significantly higher at day 28 among PRV recipients as compared to controls (Table 4). At baseline, 160 PRV recipients (54.8%) and 165 controls (56.5%) had IgA levels of <20 U/mL, and 91 (31.1%) and 92 (31.4%), respectively, had IgG levels of <20 U/mL. Among these IgA-seronegative participants, 91 (56.9%) in the PRV group and 52 (31.5%) in the control group had day 28 levels of ≥20 U/mL (*P* < .0001). Among all tested participants, regardless of baseline status, 44.9% of PRV recipients and 27.4% of controls experienced a ≥3-fold increase in IgA titers (*P* < .0001), and 74.7% PRV recipients and 58.9% controls had day 28 levels of ≥20 U/mL (*P* < .0001). Among all tested participants, the ratio of the day 28 anti–rotavirus IgA GMC in the PRV group to that in the control group was 1.7 (95% CI, 1.2–2.4; *P* = .0033). Serum IgG responses were more vigorous than IgA responses, and as with IgA, responses were significantly higher among PRV recipients as compared to controls (Table 4).

**Table 4.** Anti–Rotavirus Immunoglobulin A (IgA) and Immunoglobulin G (IgG) Responses in Infants, by Vaccines Received—Per-Protocol Population

Variable	MV, YFV, MenAV, and PRV		MV, YFV, and MenAV		Difference, Percentage Points	*P*
	Proportion	Percentage (95% CI)	Proportion	Percentage (95% CI)		
Level						
IgA						
≥3-fold increase from baseline to day 28	131/292	44.9 (39.2–50.6)	80/292	27.4 (22.3–32.5)	17.5	<.0001
≥20 U/mL						
Overall	218/292	74.7 (69.7–79.6)	172/292	58.9 (53.3–64.5)	15.8	<.0001
Baseline level <20 U/mL	91/160	56.9 (49.2–64.5)	52/165	31.5 (24.4–38.6)	25.4	<.0001
IgM						
≥3-fold increase from baseline to day 28	168/293	57.3 (51.7–63.0)	77/293	26.3 (21.2–31.3)	31.1	<.0001
≥20 U/mL						
Overall	275/293	93.9 (91.1–96.6)	223/293	76.1 (71.2–81.0)	17.7	<.0001
Baseline level <20 U/mL	76/91	83.5 (75.9–91.1)	29/92	31.5 (22.0–41.0)	52.0	<.0001
Time point	GMC (95% CI)		GMC (95% CI)		GMC Ratio	
IgA						
Baseline	25.3 (19.7–32.6)		23.7 (18.6–30.3)		…	
Day 28	118.4 (90.9–154.3)		67.9 (49.9–92.3)		1.7	.0033
IgG						
Baseline	62.5 (49.6–78.8)		58.9 (47.1–73.7)		…	
Day 28	363.6 (293.6–450.4)		153.3 (113.8–206.5)		2.3	<.0001

Abbreviations: CI, confidence interval; GMC, geometric mean concentration; MenAV, meningococcal A conjugate vaccine; MV, measles vaccine; PRV, pentavalent rotavirus vaccine; YFV, yellow fever vaccine.

### Serotype A Meningococcus

Serum meningococcal A bactericidal responses were available for 292 PRV recipients and 293 controls. A 4-fold rise in bactericidal titer was observed in 273 PRV recipients (93.5%) and 276 controls (94.2%), with a difference of −0.7% (95% CI, −5.2%–3.8%; Table 5 and Figure 2). Postvaccination titers of ≥8 and ≥128 also met noninferiority criteria (Table 5 and Figure 2). The ratio of the day 28 SBA GMT in the PRV group to that in the control group was 0.9 (95% CI, .7–1.3; *P* = .6709).

**Table 5.** Meningococcus Serotype A Serum Bactericidal Responses in Infants, by Vaccines Received, Per-Protocol Population

Variable	MV, YFV, MenAV, and PRV		MV, YFV, and MenAV		Difference, Percentage Points (95% CI)
	Proportion	Percentage (95% CI)	Proportion	Percentage (95% CI)	
Serologic finding					
Seroresponse^a^	273/292	93.5 (90.7–96.3)	276/293	94.2 (91.5–96.9)	−0.7 (−5.2–3.8)
Bactericidal titer					
≥8	282/292	96.6 (94.5–98.7)	282/293	96.2 (94.1–98.4)	0.3 (−3.8–4.4)
≥128	276/292	94.5 (91.9–97.1)	276/292	94.2 (91.5–96.9)	0.3 (−4.1–4.8)
Time point	GMT (95% CI)		GMT (95% CI)		GMT Ratio (95% CI)
Baseline	3.22 (2.68–3.88)		2.81 (2.40–3.28)		…
Day 28	2014.25 (1626.21–2494.89)		2097.03 (1693.18–2597.20)		0.94^b^ (.69–1.26)

Abbreviations: CI, confidence interval; GMT, geometric mean titer; MenAV, meningococcal A conjugate vaccine; MV, measles vaccine; PRV, pentavalent rotavirus vaccine; YFV, yellow fever vaccine

^a^Defined as a ≥4-fold increase in titer from baseline to day 28.

^b^ *P* = .6709.

### Safety

There were no immediate reactions following vaccination. Systemic reactions occurred in 29 of 300 PRV recipients (9.7%) and 30 of 300 controls (10.0%) during the first 7 days of follow-up (*P* = 1.000). At least 1 unsolicited AE was observed through day 28 in 103 of 300 PRV recipients (34.3%) and 125 of 300 controls (41.7%; *P* = .0772). Of note, 39 of 300 PRV recipients (13.0%) and 51 of 300 controls (17.0%) experienced gastrointestinal illness (*P* = .2083) with complaints of gastroenteritis, vomiting, or diarrhea from vaccination until day 28. A total of 15 participants (7 of 300 PRV recipients [2.3%] and 8 of 300 controls [2.7%]) experienced a single SAE each over the 3-month follow-up period (*P* = 1.000). These events were considered by the investigator to be unrelated to vaccination. There were no fatal SAEs, and all resolved without sequelae. There were no cases of intussusception.

## DISCUSSION

In this first study to evaluate a booster dose of rotavirus vaccine in African infants, PRV was well tolerated and elicited robust rotavirus-specific immune responses among 9–11-month-old Malian infants. Responses to MV, YFV, and MenAV were similar regardless of whether the infant also received a booster dose of PRV. Our results met our prespecified noninferiority outcome for MV and demonstrate that PRV, when coadministered with MV, does not interfere with the immune response to MV for up to at least 3 months after vaccination. We did not meet our prespecified noninferiority criterion for YFV, as the CI for the difference between seroresponse rates in PRV and control groups was 12.5% when seroresponse was defined as a ≥4-fold increase in titer. This effect was not present when the secondary definition of seroresponse (ie, a ≥2-fold increase in titer) was used or when seroconversion was compared among those who were seronegative at baseline. Given the inclusion of MenAV into the Malian Expanded Program on Immunization schedule beginning in 2017, it is reassuring that concomitant administration of PRV did not interfere with serum bactericidal responses to that antigen.

Infants who received PRV had postvaccination increases in rotavirus-specific IgA and IgG antibody measurements that were highly statistically significant as compared to those in infants who did not receive PRV. These increases are particularly important for infants with low levels of antibody at baseline. Prior to booster vaccination, over half of participants had IgA levels of <20 U/mL, consistent with susceptibility to severe disease and indicating that vaccination could be beneficial [17]. Seroconversion rates among infants with baseline levels of <20 U/mL were superior in the PRV group and included a nearly 2-fold increase in the IgA GMC. Of note, based on IgA measurements, 31.5% of controls and 56.9% of PRV recipients seroconverted in the month between receipt of vaccine and the follow-up visit at which blood specimens were collected. Wild-type rotavirus circulated during the study period and likely influenced these results in both groups. There were more adverse events identified as gastrointestinal illness in the control group as compared to the PRV group through 1 month after vaccination, although the difference was not statistically significant.

Our results are similar to those from a prior study of a booster dose of oral monovalent human rotavirus vaccine, conducted among 9-month-old children in Bangladesh. In that study, rotavirus vaccine was well tolerated, and the proportion of infants with a protective immune response to anti–rotavirus IgA and IgG was significantly higher among infants who received the booster [8]. As with the current study, there was no evidence of interference with MV in the Bangladesh study. While data from these booster dose studies support that administration of a PRV dose at 9 months of age could enhance and extend protection in infants, a correlation between individual immune responses to rotavirus vaccines and protection from rotavirus disease has not been established. Data from clinical trials show that, on average, mean levels of anti–rotavirus IgA in a population are related to the population-level efficacy against severe disease [18, 19]. Because IgG antibodies may be maternally derived, they are generally not used in young infants to evaluate immune responses. However, as in this study, IgG titers may be useful measurements in older children and adults. Properly designed field studies of the quality required to inform policy are needed to establish the effect of a booster dose on rotavirus disease outcomes.

Initially, concerns about the association between oral rotavirus vaccines and intussusception led to age restrictions on the administration of rotavirus vaccines. The World Health Organization reviewed data on the risk and benefits of rotavirus vaccines in 2012 and concluded that the additional lives saved by removing age restrictions would far outnumber excess vaccine-associated intussusception morbidity and mortality [20, 21]. While this recommendation was made in the context of the primary immunization schedule, additional data may allow the same rationale to be applied to a booster dose. This study generated relevant safety data regarding use of PRV at later ages. The vaccine was well tolerated, and adverse events were observed similarly in both groups. No cases of intussusception were reported in either group, although this study was underpowered to assess that rare outcome.

This study was the first to evaluate the serum bactericidal response when MenAV is administered with PRV. Mali was one of the first countries to introduce MenAV, via widespread campaigns in 2010 and 2011 [22]. Since then, meningococcal A disease has dramatically declined in the region [23], and since 2017, a 5-µg dose of MenAV has been used in the routine immunization program at 9 months of age to protect new cohorts of children. Our results demonstrate that seroresponse rates were equally robust in both groups and that there was no interference by PRV. These findings are consistent with those observed in a study of a meningococcus serotype C conjugate vaccine [24].

Yellow fever continues to be a risk in the African region [25], and infant vaccination and maintaining population immunity remain public health priorities. While the clinical significance of failing to meet our predefined noninferiority criteria is uncertain, the overall lower-than-expected antibody responses to YFV in both groups are concerning. In a previous study of YFV and MenAV coadministration in Malian infants, >95% of infants in every group demonstrated an NT of >1:8 [26], compared with approximately 75% in our study. Both studies used vaccine from the same manufacturer, but differences in the potency of the vaccine lot, improper cold chain handling, or performance of the NT assay could have contributed to the lower responses in the current study. The prior study identified inferior immune responses to YFV when the vaccine is coadministered with a 5-µg dose of MenAV, as used here. Mutual interference was also found between YFV and a measles-mumps-rubella vaccine in Brazil [27]. The combination of this previous work with our results suggests that further studies to evaluate the kinetics and magnitude of antibody responses to yellow fever virus are warranted.

The burden of severe rotavirus gastroenteritis remains high in Mali and other resource-poor countries in the second year of life [2, 6, 7, 28]. This study of a booster dose of rotavirus vaccine, the first to be conducted in African infants, strongly supports that a PRV booster dose strategy is feasible, well tolerated, and immunogenic. While these results are compelling, global policymakers will need strong clinical evidence along with cost-effectiveness and expected impact data to recommend immunization schedule changes. In the absence of an immunological correlate of protection, efficacy studies are urgently needed to determine if an additional dose of rotavirus vaccine will be a safe and effective strategy to extend protection from the primary series into the second year of life.

## Notes

***Acknowledgments.*** We thank the participants and their families for the generous contribution of their time and effort.

The corresponding author had full access to all the data in the study and had final responsibility for the decision to submit for publication.

***Disclaimer.*** The Bill and Melinda Gates Foundation and PATH had no role in study design, data collection, data analysis, data interpretation, or writing of the report.

***Financial support.*** This work was supported by PATH and the Bill and Melinda Gates Foundation.

***Potential conflicts of interest.*** M. D. T., A. T., and K. K. receive research funds from Merck. M. F. receives research funds from the National Institutes of Health. Since the study was completed, S. O. S. has become the Mali Minister of Health. All other authors report no potential conflicts of interest. All authors have submitted the ICMJE Form for Disclosure of Potential Conflicts of Interest. Conflicts that the editors consider relevant to the content of the manuscript have been disclosed.

Presented in part: 10th African Rotavirus Symposium, Bamako, Mali, 2 June 2016; 65th Annual Meeting of the American Society of Tropical Medicine and Hygiene, Atlanta, Georgia, 15 November 2016 (abstract 1064).
