# Erratum

**DOI:** 10.1093/infdis/jiy540

**Published:** 2018-10-25

In the 15 August 2018 issue of the *Journal*, in the article by Haidara et al (Haidara FC, Tapia MD, Sow SO, et al. Evaluation of a booster dose of pentavalent rotavirus vaccine coadministered with measles, yellow fever, and meningitis A vaccines in 9-month-old Malian infants. J Infect Dis **2018**; 218:606–13), the disclosure and financial support information was incorrect. The information should read “***Disclaimer.*** The Bill and Melinda Gates Foundation had no role in study design, data collection, data analysis, data interpretation, or writing of the report.

***Financial support.*** This work was supported by the Bill and Melinda Gates Foundation (grant to PATH).”

The authors regret this error.

